# Identification of a Novel Response Regulator, Crr1, That Is Required for Hydrogen Peroxide Resistance in *Candida albicans*

**DOI:** 10.1371/journal.pone.0027979

**Published:** 2011-12-02

**Authors:** Catherine R. Bruce, Deborah A. Smith, David Rodgers, Alessandra da Silva Dantas, Donna M. MacCallum, Brian A. Morgan, Janet Quinn

**Affiliations:** 1 Institute for Cell and Molecular Biosciences, Faculty of Medical Sciences, Newcastle University, Newcastle upon Tyne, United Kingdom; 2 Aberdeen Fungal Group, Institute of Medical Sciences, University of Aberdeen, Aberdeen, United Kingdom; Louisiana State University, United States of America

## Abstract

*Candida albicans* colonises numerous niches within humans and thus its success as a pathogen is dependent on its ability to adapt to diverse growth environments within the host. Two component signal transduction is a common mechanism by which bacteria respond to environmental stimuli and, although less common, two component-related pathways have also been characterised in fungi. Here we report the identification and characterisation of a novel two component response regulator protein in *C. albicans* which we have named *CRR1* (*Candida R*esponse *R*egulator 1). Crr1 contains a receiver domain characteristic of response regulator proteins, including the conserved aspartate that receives phosphate from an upstream histidine kinase. Significantly, orthologues of *CRR1* are present only in fungi belonging to the *Candida* CTG clade. Deletion of the *C. albicans CRR1* gene, or mutation of the predicted phospho-aspartate, causes increased sensitivity of cells to the oxidising agent hydrogen peroxide. Crr1 is present in both the cytoplasm and nucleus, and this localisation is unaffected by oxidative stress or mutation of the predicted phospho-aspartate. Furthermore, unlike the Ssk1 response regulator, Crr1 is not required for the hydrogen peroxide-induced activation of the Hog1 stress-activated protein kinase pathway, or for the virulence of *C. albicans* in a mouse model of systemic disease. Taken together, our data suggest that Crr1, a novel response regulator restricted to the *Candida* CTG clade, regulates the response of *C. albicans* cells to hydrogen peroxide in a Hog1-independent manner that requires the function of the conserved phospho-aspartate.

## Introduction

Two component signal transduction is a primary mechanism utilised by bacteria to respond to environmental stimuli. These signalling modules are comprised of a sensor histidine kinase and a response regulator protein containing a receiver domain [1]. Upon stimulation, phosphate is transferred from a histidine residue in the kinase to an aspartate residue located in the receiver domain of the response regulator protein. This phosphorylation influences the activity of the response regulator protein to trigger the appropriate response to the environmental stimulus. Two component-related signal transduction mechanisms are also utilised, although less extensively, in certain eukaryotes including fungi, slime mould and plants [2]. Interestingly, in contrast to the bacterial systems, a more complex multi-step phosphorelay involving three components appears to predominate in eukaryotic systems. Such eukaryotic pathways typically consist of a hybrid sensor histidine kinase, containing both kinase and receiver domains, an intermediary phosphorelay protein and a response regulator protein containing a receiver domain. In these cases phosphate is transferred from a histidine residue in the kinase domain to an aspartate residue located in the receiver domain of the histidine kinase. This phosphate is then transferred to a histidine residue in the phosphorelay protein which then completes transfer to an aspartate residue in the receiver domain of the response regulator.

A function unique to eukaryotic two component-related signalling pathways is to relay stress signals to stress-activated protein kinase (SAPK) pathways, which are important stress signalling modules solely found in eukaryotes [3]. In the model yeast *Saccharomyces cerevisiae*, osmotic stress-induced activation of the Hog1 SAPK is regulated by a multi-step two component-related system consisting of the Sln1 histidine kinase, the Ypd1 phosphorelay protein and the Ssk1 response regulator, which functions in parallel with a second pathway that contains the Sho1 transmembrane protein [4]. In response to osmotic stress, the Sln1 histidine kinase is inactivated due to loss of turgor pressure within the membrane [5]. This subsequently halts phosphorelay through Ypd1 leading to a rapid dephosphorylation of Ssk1 [6]. Dephosphorylated Ssk1 activates the MAPKKKs Ssk2/Ssk22 [7], which subsequently activate Hog1. Interestingly, in the distantly related yeast *Schizosaccharomyces pombe*, an analogous system comprising of the histidine kinases, Mak2 (Phk1) and Mak3 (Phk2) [8], [9], the phosphorelay protein Mpr1 (Spy1) [10] and the response regulator Mcs4 [8], functions to relay hydrogen peroxide, but not osmotic, stress signals to the Hog1-related Sty1 (Spc1) SAPK pathway. Peroxide sensing by the *S. pombe* two component-related pathway is mediated by GAF and PAS domains present in the Mak2 and Mak3 kinases [9].

In addition to Ssk1/Mcs4, *S. cerevisiae* and *S. pombe* both contain a second response regulator protein termed Skn7 [11] and its homologue Prr1 [12], respectively. However, unlike Ssk1/Mcs4, the Skn7 and Prr1 response regulators are transcription factors that do not regulate the Hog1/Sty1 SAPK pathways. In *S. cerevisiae*, Skn7 regulates the expression of genes involved in the cell wall and the oxidative stress response [13], [14] yet, interestingly, two component-mediated phosphorylation of Skn7 is only required for the cell wall functions of this transcription factor [13], [14]. In contrast, recent studies illustrated that Prr1 is required for the transcriptional response of *S. pombe* cells to a wide range of hydrogen peroxide concentrations [9], [15] and that two component-mediated phosphorylation of Prr1 is required for the response to high but not low levels of hydrogen peroxide [9].

Two component proteins, related to those in *S. cerevisiae* and *S. pombe*, have also been identified in the major fungal pathogen of humans, *Candida albicans* [16]. Stress responses are intimately linked with the virulence of this medically important fungus [17], and notably several of these two component proteins have been implicated in pathogenesis [16]. *C. albicans* contains three structurally distinct histidine kinases; Sln1 is most similar to the Sln1 osmosensor in *S. cerevisiae* [18], Chk1 is the closest homologue of the Mak2 and Mak3 hydrogen peroxide stress sensors in *S. pombe* [19], and Nik1/Cos1 is related to the Nik-1 histidine kinase in *Neurospora crassa* [18], [20], [21]. *C. albicans* also contains a single phosphorelay protein, Ypd1 [22], and homologues of the Ssk1 and Skn7 response regulators [23], [24]. Indeed, similar to Ssk1 and Mcs4 in *S. cerevisiae* and *S. pombe*, respectively, Ssk1 is important for the regulation of the Hog1 SAPK in *C. albicans*. Specifically, Ssk1 is required for efficient oxidative stress-induced activation of Hog1 in *C. albicans* [25], [26], which is reminiscent of Mcs4 regulation of the Sty1 SAPK in *S. pombe*. However, the identity of the histidine kinase(s) responsible for sensing and signalling oxidative stress signals to Ssk1 in *C. albicans* remains elusive [27], [28]. *C. albicans* also contains Skn7, a homologue of the Skn7/Prr1 response regulators in *S. cerevisiae* and *S. pombe* and, similar to findings in these model yeasts, *C. albicans* cells lacking Skn7 display impaired resistance to oxidative stress-inducing agents [24].

Here, we describe the identification and characterisation of a novel response regulator in *C. albicans*, which we name Crr1 (*Candida R*esponse *R*egulator 1), that is not conserved in *S. cerevisiae* or *S. pombe*. We demonstrate that Crr1 is specifically involved in the response of *C. albicans* to hydrogen peroxide stress, but not to other oxidising agents or a range of other stress conditions. Furthermore, our data suggests that Crr1 functions in a Hog1-independent pathway and that the role of the protein in hydrogen peroxide responses is regulated by the phosphorylation of the conserved aspartic acid residue within the receiver domain. Collectively, our data suggests that the novel response regulator protein Crr1 functions in a hitherto unidentified two component signal transduction pathway to specifically regulate the response of *C. albicans* to hydrogen peroxide.

## Materials and Methods

### Ethics statement

The animal experiments carried out were approved by the University of Aberdeen local ethical review committee and under Project Licence PPL 60/4135, granted by the UK Home Office. All work conformed to UK Home Office regulations.

### Strains and growth conditions

The *C. albicans* strains used in this study are listed in Table 1. Cells were grown at 30°C in either YPD media (2% yeast extract, 1% bactopeptone, 2% glucose) or SD media (6.79 g/l yeast nitrogen base without amino acids, 2% glucose) supplemented with the required nutrients for auxotrophic mutants [29].

**Table 1:** Strains used in this study.

Strain	Genotype	Source
RM1000	*ura3::λ imm434/ura3::λimm434, his1::hisG/his1::hisG*	[51]
JC50	RM1000 *hog1::LoxP-ura3-LoxP, hog1::LoxP-HIS1-LoxP*+CIp20	[36]
JC52	RM1000 *hog1::LoxP-ura3-LoxP, hog1::LoxP-HIS1-LoxP*+CIp20-*HOG1*	[36]
JC806	RM1000+CIp20	This study
JC528	RM1000 *crr1::hisG/crr1::hisG*	This study
JC566	RM1000 *crr1::hisG/crr1::hisG*+CIp20	This study
JC803	RM1000 *crr1::hisG/crr1::hisG*+CIp20-*CRR1*	This study
JC804	RM1000 *crr1::hisG/crr1::hisG*+CIp20-*CRR1*	This study
JC784	RM1000 *ssk1::LoxP-URA3-LoxP/ssk1::LoxP-HIS1-LoxP*	This study
JC787	RM1000 *crr1::hisG/crr1::hisG, ssk1::LoxP-URA3-LoxP/ssk1::LoxP-HIS1-LoxP*	This study
JC924	RM1000 *crr1::hisG/crr1::hisG*+pACT1-*CRR1GFP*	This study
JC926	RM1000 *crr1::hisG/crr1::hisG*+pACT1-*CRR1(D209N)GFP*	This study
SN148	*arg4Δ*/*arg4*Δ *leu2Δ*/*leu2Δ his1Δ*/*his1Δ ura3Δ::imm434*/*ura3Δ::imm434 iro1Δ::imm434/iro1Δ::imm434*	[32]
JC747	SN148+CIp30	[52]
JC1552	SN148 *ssk1::LoxP-ARG4 -LoxP/ssk1::LoxP-HIS1-LoxP*+CIp20	This study
JC1571	SN148 *crr1::LoxP-ARG4 -LoxP/crr1::LoxP-HIS1-LoxP*	This study
JC1572	SN148 *crr1::LoxP-ARG4 -LoxP/crr1::LoxP-HIS1-LoxP*+CIp20	This study
JC1574	SN148 *crr1::LoxP-ARG4 -LoxP/crr1::LoxP-HIS1-LoxP*+CIp20-*CRR1*	This study
JC1576	SN148 *crr1::LoxP-ARG4 -LoxP/crr1::LoxP-HIS1-LoxP+*pACT1-*CRR1GFP*	This study
JC1578	SN148 *crr1::LoxP-ARG4 -LoxP/crr1::LoxP-HIS1-LoxP+*pACT1-*CRR1(D209N)GFP*	This study

### Strain construction

The oligonucleotide primers used for generating the constructs described below are listed in Table 2.

**Table 2:** Oligonucleotides used in this study.

Name	Sequence 5′ to 3′
CRR1PromF	gcgcggatccgcgaaagttcacagttattgtg
CRR1TermR	gcgcggatcctataaacacgacaaacctccttgg
CRR1delF	aaattgcctccccctgttgcaagtaatttttcctcctttttttttgatttgtatatttttacaaccaataagttattattgaattcattgtacacactaaccagggttttcccagtcacg
CRR1delR	aaacatcgtagaacaacgtagaaacaaccataaaccattcaaagaaacaagatacaaaacaaaaatataagtcaaacaaaaaacccgctctgaatgcatctcactaaagggaacaaaagc
SSK1delF	ctaggggaaccaaaaaaaaaaatattaaaaataaccaagaaagaaataaagaaacaagaattctgcttataaaacgaatataaaaaaaaaataataactcccagggttttcccagtcacg
SSK1delR	aattttatcaatcattaaaagcaaaaactgaaaaaaaccgaaaacctaatttattccaacgactcatcttagtggcatttcataaatccgtttttttcttctcactaaagggaacaaaagc
CRR1HindIIIF	cggcccaagcttatgatatccatgaacccaattatg
CRR1GFPHindIIIR	cggcccaagcttgctattttgttttttcttg
CRR1DNmutF	cattccatatttatcaacattgagatgcctgatg
CRR1MHR	gaattcgctagcttaatgatggtgatgatggtgaagtcctcctcgctgatcaatttttgttcttcagccatggacaaatcttcttcagaaattaacttttgctcctctattttgttttttcttgttataattatatc
CRR1PstIF	aatgtctgcagccatcaatcggtatataatttggaag

#### Deletion of CRR1

The *CRR1* locus was disrupted by Ura-blasting [30] in RM1000 to generate strain JC528 (*crr1Δ*). The *crr1::hisG-URA3-hisG* disruption cassette deleted codons 2–281 of the 282 codon predicted open reading frame. Gene disruptions were confirmed by PCR. To construct re-integrant control strains the *CRR1* gene plus 1000 bp of the promoter region and 214 bp of the terminator region were amplified by PCR, using the oligonucleotide primers CRR1PromF and CRR1TermR, and ligated into the *Bam*HI site of CIp20 to create Clp20-*CRR1* [31]. The CIp20-*CRR1* plasmid was digested with *Stu*I and integrated at the *RPS10* locus in the *crr1Δ* mutant to generate strains JC803 and JC804. To generate a *crr1Δ* deletion mutant that was auxotrophically identical to the reconstituted strain, the CIp20 vector was integrated at the *RPS10* locus in the *crr1Δ* mutant to generate strain JC566. *CRR1* was also deleted in a second strain background, SN148 [32]. *CRR1* disruption cassettes, comprising either the *ARG4* gene or the *HIS1* gene flanked by *loxP* sites and 100 nucleotides corresponding to regions 5′ and 3′ of the *CRR1* open reading frame, were generated by PCR using the oligonucleotide primers CRR1delF and CRR1delR and the plasmid templates pLAL2 or pLHL2 [33], respectively. These *CRR1* disruption cassettes replaced the entire 282 codon open reading frame of *CRR1*. To construct the re-integrant control strain, the CIp20-*CRR1* plasmid was digested with *Stu*I as above and integrated at the *RPS10* locus in the *crr1Δ* mutant (JC1571) to generate strain JC1574. As above, a *crr1Δ* deletion mutant that was auxotrophically identical to the reconstituted strain was generated by integrating the CIp20 vector at the *RPS10* locus in the *crr1Δ* mutant to generate strain JC1572.

#### Deletion of SSK1

*SSK1* disruption cassettes, comprising either the *URA3* gene or *HIS1* gene flanked by *loxP* sites and 100 nucleotides of DNA sequence corresponding to regions 5′ and 3′ of the *SSK1* open reading frame, were generated by PCR using the oligonucleotide primers SSK1delF and SSK1delR, and the plasmid templates pLUL2 or pLHL2, respectively [33]. These *SSK1* disruption cassettes, which deleted the entire 674 codon open reading frame, were sequentially introduced into *C. albicans* RM1000 (*CRR1*) or *crr1Δ* (JC528) cells to disrupt both alleles of *SSK1* and generate strains JC784 and JC787, respectively. Gene disruptions were confirmed by PCR. *SSK1* was also deleted in a second strain background, SN148 [32]. *SSK1* disruption cassettes, comprising either the *ARG4* gene or the *HIS1* gene flanked by *loxP* sites and 100 nucleotides corresponding to regions 5′ and 3′ of the *SSK1* open reading frame, were generated by PCR using the oligonucleotide primers SSK1delF and SSK1delR and the plasmid templates pLAL2 or pLHL2 [33], respectively. The CIp20 vector was integrated at the *RPS10* locus in the resulting *ssk1Δ* mutant to generate strain JC1552.

#### GFP-tagging and mutagenesis of Crr1

To tag Crr1 at the C-terminus with GFP, the *CRR1* gene was amplified by PCR using the oligonucleotide primers CRR1HindIIIF and CRR1GFPHindIIIR, and ligated into the *Hin*dIII site adjacent to the GFP sequence in pACT1-GFP [34] to create pACT1-*CRR1GFP*. The pACT1-*CRR1GFP* plasmid was linearised by digestion with *Stu*I to target integration at the *RPS10* locus in *C. albicans crr1::hisG/crr1::hisG* (JC528) and *crr1::HIS1/crr1::ARG4* (JC1571) cells. In the resulting strains, JC924 and JC1576 respectively, expression of *CRR1GFP* is controlled by the *ACT1* promoter. Mutagenesis of *CRR1* to create the *crr1^D209N^* allele was performed by a two-stage PCR in which a mega primer, generated using the oligonucleotides CRR1DNmutF and CRR1MHR and the plasmid CIp10-*CRR1* as template, was subsequently used with the oligonucleotide CRR1PstIF and the plasmid CIp10-*CRR1* as template. The resulting 1.2 kb PCR fragment was digested with *Pst*I and *Nhe*I and ligated into CIp-C-ZZ [35] digested with *Pst*I and *Nhe*I to remove the TEV-protein A-encoding sequence. The resulting plasmid CIp-*CRR1(D209N)* was used as template for PCR, using the oligonucleotide primers CRR1HindIIIF and CRR1GFPHindIIIR, and the PCR fragment produced was then ligated into the *Hin*dIII site of pACT1-GFP as above. The resulting pACT1-*CRR1(D209N)GFP* plasmid was linearised and targeted to the *RPS10* locus in *C. albicans crr1::hisG/crr1::hisG* (JC528) and *crr1::HIS1/crr1::ARG4* (JC1571) cells, as described above, to generate strains JC926 and JC1578, respectively. The integrated open reading frames and the correct chromosomal insertion of the GFP-tagged derivatives of *CRR1* were confirmed by PCR and DNA sequencing.

### Stress sensitivity tests

*C. albicans* strains to be tested were grown in liquid culture at 30°C to exponential phase and then 10 fold serial dilutions were spotted onto YPD plates containing the indicated compounds, using a 48-well replica plater (Sigma-Aldrich). Plates were incubated at 30°C for 24 h.

### Hog1 phosphorylation assays

Cells were grown to mid-exponential phase at 30°C and exposed to either 5 mM hydrogen peroxide or 1 M NaCl for the indicated times. Protein extracts were prepared and phosphorylated Hog1 was detected by western blot with an anti-phospho-p38 antibody (New England Biolabs) as described previously [36]. Blots were stripped and total levels of Hog1 were determined by probing with an anti-Hog1 antibody (Santa Cruz Biotechnology).

### Microscopy

Cells were fixed in 3.7% para-formaldehyde, washed in PEM (100 mM PIPES pH 7.6, 1 mM EGTA, 1 mM MgSO_4_) and spread onto poly-L-lysine-coated slides as described previously [36]. Cover slips were mounted onto slides using Vectashield mounting medium containing DAPI (Vector Laboratories, Burlingame, CA). DAPI and GFP fluorescence were captured by exciting cells with 365 nm and 450–490 nm wavelengths, respectively, using a Zeiss Axioscope microscope, with a 63× oil immersion objective, and Axiovision imaging system.

### Virulence analysis

The standard 28-day survival method was employed to examine the potential role of Crr1 in mediating *C. albicans* virulence. Female BALB/c mice (6–8 weeks; Harlan, UK) were housed in groups of 6 with food and water provided *ad libitum*. *C. albicans* strains RM1000+CIp20 (JC806), *crr1Δ*+CIp20 (JC566), and *crr1Δ*+CIp20-*CRR1* (JC803), were grown in NGY medium (0.1% neopeptone, 0.4% glucose, 0.1% yeast extract) for 18 h at 30°C with constant agitation. Cells were harvested in sterile saline, washed twice, and resuspended to produce inoculants containing approximately 2.5×10^6^ cfu/ml. Mice were injected with 100 µl of each strain, with inoculants ranging from 1.2–1.4×10^4^ cfu/g mouse body weight. All experimental work was carried out under UK Home Office licence regulations and conformed to the requirements of the Ethical Review Committee of the University of Aberdeen. Mouse condition and weight were monitored daily, with mice culled either when they showed signs of severe infection or if weight decreased by more than 20% from the initial body weight. For all culled mice, death was recorded as occurring on the following day. At the time of death, the left kidney and spleen were aseptically removed and homogenised in saline for organ burden determination. Mouse survival was plotted and compared by Kaplan-Meier survival plots and kidney/spleen counts compared by Kruskall-Wallis non-parametric test.

## Results

### Identification of a novel response regulator in *C. albicans*

**Figure 1:**
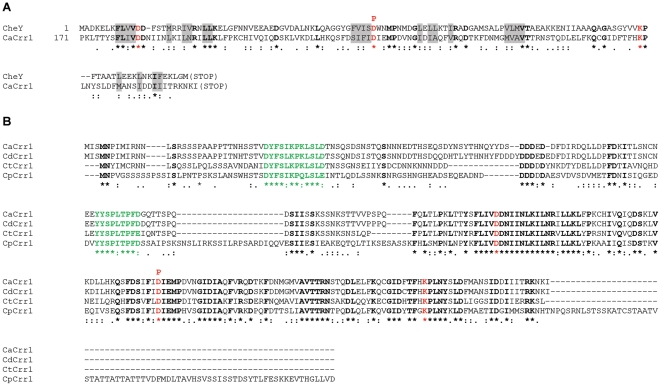
Sequence analysis of the response regulator protein Crr1 in *C. albicans* and identification of homologues in other diploid members of the *Candida* CTG clade. (A) Clustal alignment of the receiver domain located in the C-terminal region of Crr1 (*orf19.5843*) of *C. albicans* (CaCrr1) and the CheY response regulator protein of *Escherichia coli* which essentially consists of a receiver domain. Residues that are identical between the receiver domains are indicated by bold, the aspartate and lysine residues conserved in all receiver domains are shown in red bold, and the aspartate residue which is predicted to be phosphorylated by two component signal transduction by a bold red “P”. Hydrophobic residues that are components of the hydrophobic core of CheY are indicated by grey shading. Note that the homology between the receiver domains extends to the replacement of amino acids with others with similar chemical properties. A colon indicates a highly similar substitution and a full stop a similar substitution. (B) Clustal alignment of potential Crr1 homologues in *C. albicans* (CaCrr1), *C. dubliniensis* (CD36_30940; CdCrr1), *C. tropicalis* (CTRG_00590; CtCrr1), and *C. parapsilosis* (CPAG_04104; CpCrr1). Residues shared by all four proteins are highlighted as described in (A) above. Two conserved regions were identified (green bold) that lie N-terminal to the potential receiver domain in each protein. The predicted protein sequences of the Crr1 homologues in the diploid members of the *Candida* CTG clade were obtained by BLAST analyses at the *C. albicans* genome web site (http://candidagenome.org/).

To shed more insight into the roles of two component signal transduction pathways in *C. albicans* we analysed the genome database (http://www.candidagenome.org/) for potential hitherto unidentified two component signal transduction proteins. This analysis revealed an uncharacterised open reading frame (*orf19.5843*) in *C. albicans* which we have named *CRR1* (*Candida R*esponse *R*egulator 1), that encodes a potential novel two component response regulator protein. Analysis of the predicted sequence of Crr1 revealed a potential receiver domain, that contains all the key residues found in such domains, including two highly conserved aspartate residues, one of which receives phosphate from an upstream histidine kinase, and a highly conserved lysine residue (Fig. 1A, indicated in red bold). Moreover, the similarity of the potential receiver domain of Crr1 to the prototypical bacterial response regulator CheY, extends to a large number of hydrophobic residues that are components of the hydrophobic core of CheY (Fig. 1A, indicated in grey shading; [37]). Interestingly, homologues of Crr1 are not found in either of the well characterised model yeasts, *S. cerevisiae* or *S. pombe*. Indeed, the only closely related homologues of Crr1 are encoded by uncharacterised open reading frames present in other members of the *Candida* CTG clade [38] (Figs. 1B; S1). Moreover, it is noteworthy that the homology to Crr1 extends outside of the receiver domain only in the diploid members of the *Candida* CTG clade [38]; *Candida dubliniensis*, *Candida tropicalis*, *Candida parapsilosis* and *Lodderomyces elongisporus* (Fig. 1B and data not shown), whereas significant homology is restricted to the receiver domain in members of the haploid subclade [38]; *Debaromyces hansenii*, *Candida guilliermondii* and *Candida lusitaniae* (Fig. S1). It is also interesting to note that sequence analysis of the proteins in the haploid subclade did not reveal any obvious homology outside of the potential receiver domain within this subgroup of proteins (Fig. S1B). Taken together our analysis has revealed a novel family of response regulator proteins that appears to be confined to the *Candida* CTGclade.

### Crr1 is required for hydrogen peroxide resistance in *C. albicans*

**Figure 2:**
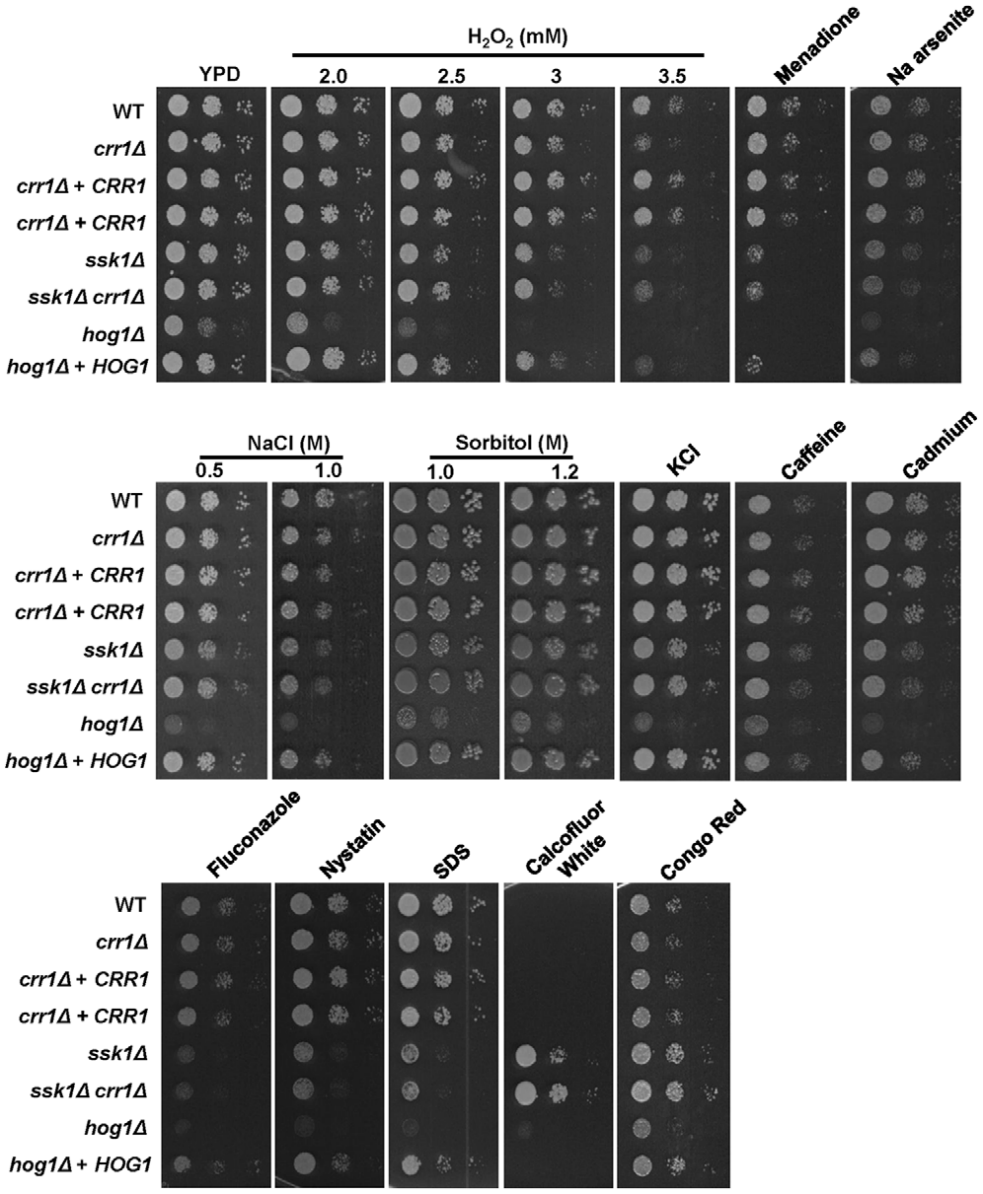
The Crr1 response regulator is required for the resistance of cells to hydrogen peroxide. 2×10^3^ cells, and 10-fold dilutions thereof, of exponentially-growing wild-type (WT, JC806), *crr1Δ* (JC566), *crr1Δ*+*CRR1* (JC803, JC804), *ssk1Δ* (JC784), *ssk1Δ/crr1Δ* (JC787), *hog1Δ* (JC50) and *hog1Δ*+*HOG1* (JC52) strains were spotted onto YPD plates containing the following agents; hydrogen peroxide (2, 2.5, 3, 3.5 mM), 250 mM menadione, 2.5 mM Na arsenite, NaCl (0.5, 1.0 M), sorbitol (1.0, 1.2 M), 0.6 M KCl, 12.5 mM caffeine, 1 mM cadmium, 5 µg/ml fluconazole, 5 µg/ml nystatin, 0.02% SDS, 50 µg/ml Calcofluor White and 200 µg/ml Congo Red. Plates were incubated at 30°C for 24 h.

To examine the function(s) of Crr1 in *C. albicans*, a homozygous null mutant was generated. Each of the two copies of the *CRR1* allele in this diploid fungus was inactivated using the ura-blaster gene disruption system, which deleted codons 2–281 of the predicted 282 codon reading frame in strain RM1000. Previous studies in *C. albicans* have implicated the other response regulator proteins, Ssk1 and Skn7, in the oxidative stress response. For example, cells lacking *SSK1* display increased sensitivity to a range of oxidative stress-inducing agents including hydrogen peroxide, menadione and potassium superoxide [25], whilst cells lacking *SKN7* display increased sensitivity to hydrogen peroxide and *t*-BOOH but not to menadione or potassium superoxide [24]. Hence, to examine the potential role of Crr1 in the response of *C. albicans* to oxidative and other stress conditions, we compared the sensitivity of wild-type, *crr1Δ*, and reintegrant (*crr1Δ*+*CRR1*) cells to an extensive panel of stress-inducing agents (Fig. 2). Deletion of *CRR1* did not impair the growth of *C. albicans* under non-stress conditions. Notably, however, deletion of *CRR1* specifically resulted in impaired resistance to the oxidative stress-inducing agent hydrogen peroxide and, importantly, this phenotype was reversed upon reintroduction of the wild-type *CRR1* gene into the *crr1Δ* strain (Fig. 2). In contrast, no notable increase in stress sensitivity was observed in response to other oxidative stress-inducing agents, such as menadione, a variety of osmotic stress-inducing agents, such as NaCl, KCl or sorbitol, heavy metals such as cadmium or arsenic, caffeine, or antifungal drugs such as fluconazole or nystatin (Fig. 2). Deletion of *CRR1* in the SN148 [32] background replicated such findings (Fig. S2A).

In *C. albicans* the Hog1 SAPK is activated in response to a range of stress conditions, including hydrogen peroxide and, moreover, the Ssk1 response regulator plays an important role in the relay of hydrogen peroxide signals to Hog1 [25], [26], [28]. In *S. cerevisiae*, the analogous Ssk1 response regulator has been shown to relay osmotic, but not oxidative, stress signals to the Hog1 SAPK and it does this in parallel with a second, Sho1-mediated, osmosensing pathway (reviewed in [4]). Intriguingly, although the analogous Sho1 pathway does not appear to relay osmotic stress signals to Hog1 in *C. albicans* [28], both *C. albicans* single *ssk1Δ* or double *ssk1Δsho1Δ* mutants retain wild-type levels of Hog1 activation following osmotic stress [25], [28]. Thus, there is a distinct mechanism of Hog1 activation in response to osmotic stress in *C. albicans* that is independent of both Sho1 and Ssk1. The identification of Crr1 raised the possibility that this novel response regulator functions redundantly with Ssk1 to relay osmotic and possibly other stress signals to the Hog1 SAPK. To investigate this hypothesis, first a double *ssk1Δcrr1Δ* mutant was created and the stress sensitive phenotypes exhibited by *ssk1Δ*, *crr1Δ*, *ssk1Δcrr1Δ* and *hog1Δ* mutants compared. Significantly, the single *ssk1Δ* and *crr1Δ* mutants displayed a similar level of sensitivity to hydrogen peroxide, intermediate to that displayed by *hog1Δ* cells, and this was not further increased in the double *ssk1Δcrr1Δ* mutant (Fig. 2). Cells lacking *SSK1* also displayed intermediate sensitivity to a range of other stress conditions such as the superoxide generator menadione, heavy metals, SDS and various drugs compared to that exhibited by *hog1Δ* cells (Fig. 2). However, deletion of *CRR1* did not result in increased sensitivity to any of these conditions either in the presence or absence of *SSK1* (Fig. 2). Similarly, whilst deletion of *SSK1* increased the resistance of cells to cell wall damaging agents as previously reported [39], such as the cell wall biogenesis inhibitors Calcofluor White and Congo Red, this was not exacerbated in the *ssk1Δcrr1Δ* double mutant (Fig. 2). Collectively, these data suggest that, although Ssk1 is involved in the response to multiple stress conditions, Crr1 is specifically required for the response of cells to hydrogen peroxide.

### Ssk1, but not Crr1, regulates Hog1 phosphorylation in response to hydrogen peroxide but both response regulators are dispensable for NaCl-induced Hog1 phosphorylation

**Figure 3:**
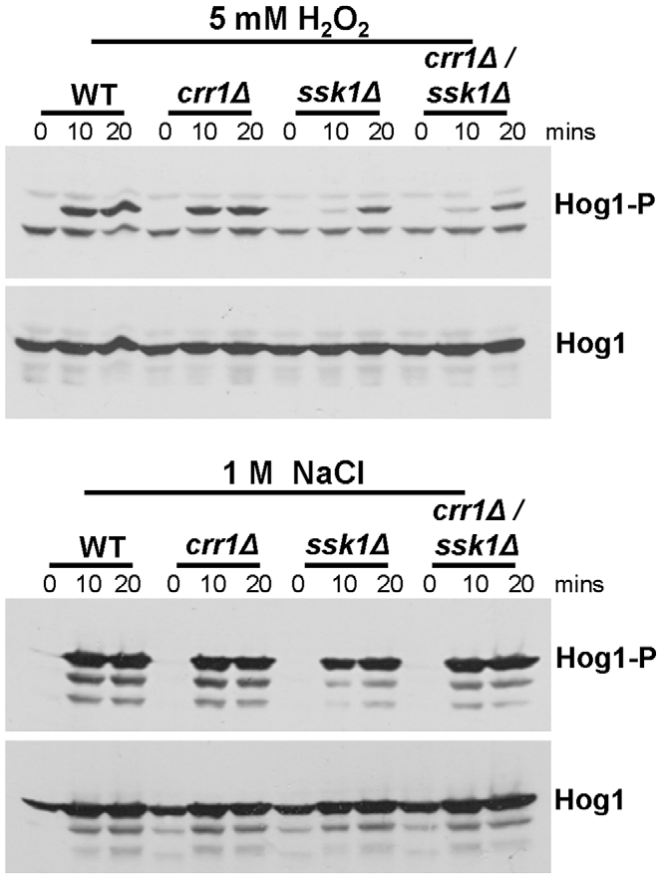
The Ssk1, but not the Crr1, response regulator is required for Hog1 activation in response to hydrogen peroxide. Western blot analysis of whole cell extracts isolated from wild-type (WT, JC806), *crr1Δ* (JC566), *ssk1Δ* (JC784), and *crr1Δssk1Δ* (JC787) cells after treatment with 5 mM hydrogen peroxide or 1 M NaCl for the specified times. Western blots were probed with an anti-phospho-p38 antibody, which specifically recognises the phosphorylated, active form of *C. albicans* Hog1 (Hog1-P). Total levels of Hog1 protein were determined by stripping the blot and reprobing with an anti-Hog1 antibody which recognises both phosphorylated and unphosphorylated forms of Hog1 (Hog1).

As cells lacking the Crr1 response regulator displayed increased sensitivity to hydrogen peroxide, in a manner that links the protein to Ssk1 function, we next examined whether, like Ssk1 [25], Crr1 relays oxidative stress signals to the Hog1 SAPK. Consistent with previous reports [25], [28], western blot analysis revealed that hydrogen peroxide-induced activation of the Hog1 SAPK was impaired in cells lacking Ssk1 (Fig. 3). However, in contrast, wild-type levels of hydrogen peroxide-induced Hog1 phosphorylation were observed in *crr1Δ* cells (Figs. 3; S2B), and the level of hydrogen peroxide-induced Hog1 activation associated with loss of *SSK1* was not further impaired in *ssk1Δcrr1Δ* double mutant cells (Fig. 3). Furthermore, consistent with the wild-type levels of osmotic stress resistance exhibited by *ssk1Δ*, *crr1Δ* and *ssk1Δcrr1Δ* cells (Figs. 2; S2A), Hog1 activation was not impaired in any of these mutants following NaCl treatment (Figs. 3; S2B).

Taken together, our data show that, whilst both response regulators Ssk1 and Crr1 are important for the oxidative stress response in *C. albicans*, Crr1 influences the response of cells to hydrogen peroxide in a pathway that is independent of Hog1 phosphorylation. Moreover, together with the observations that *ssk1Δcrr1Δ* cells display wild-type levels of osmotic stress resistance and osmotic stress-induced Hog1 activation, these data suggest that Ssk1 and Crr1 do not function redundantly to regulate Hog1 activation in response to osmotic stress.

### Mutation of the putative phospho-aspartate of Crr1 does not impact on the cellular localisation of Crr1, but does result in impaired resistance to hydrogen peroxide

**Figure 4:**
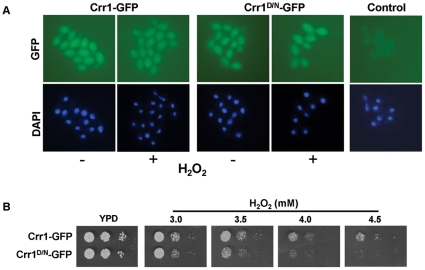
Mutation of the putative phospho-aspartate of Crr1 impairs hydrogen peroxide resistance, but does not affect the cellular localisation of the protein. (A) The localisation of GFP-tagged wild-type Crr1 (Crr1-GFP) and mutant Crr1, in which the putative phospho-aspatate residue in the receiver domain was mutated to asparagine (Crr1^D/N^-GFP), were determined by fluorescence microscopy of JC924 (Crr1-GFP) and JC926 (Crr1^D/N^-GFP) cells before (−) and after (+) treatment with 5 mM hydrogen peroxide (GFP) for 10 min. Nuclei were visualised by DAPI staining (DAPI). The control panel illustrates the level of background fluorescence observed in wild-type cells (JC806) expressing untagged Crr1. (B) 10^3^ cells, and 10-fold dilutions thereof, of exponentially-growing *crr1Δ* cells expressing either *CRR1-GFP* (JC924) or *CRR1^D/N^-GFP* (JC926) were spotted onto YPD plates containing the indicated concentrations of hydrogen peroxide and incubated at 30°C for 24 h.

Previous studies in the model yeasts *S. cerevisiae* and *S. pombe* revealed that, whilst the Ssk1/Mcs4 response regulators are cytoplasmic [9], [40], the Skn7/Prr1 response regulator transcription factors are predominantly nuclear [9], [40]. Hence, to further characterise the novel response regulator Crr1 in *C. albicans* the cellular location of a Crr1-GFP fusion protein was determined by fluorescence microscopy. To facilitate this analysis a strain was created in which a *CRR1-GFP* fusion gene was expressed from the *ACT1* promoter. This was necessary as previous experiments using epitope-tagged Crr1-fusions expressed at the *CRR1* locus, indicated that Crr1 is a very low abundance protein (unpublished obs.). In addition, to investigate whether two component-mediated phosphorylation of Crr1 may impact on either the localisation and/or function of this response regulator, the conserved aspartic acid (D209) residue located in the receiver domain, that is predicted to be phosphorylated by two component phosphorelay (Fig. 1) was substituted with asparagine (Crr1^D/N^) which is predicted to mimic hypo-phosphorylation [41]. The Crr1-GFP and Crr1^D/N^-GFP fusion proteins were found to be present in both the cytoplasmic and nuclear compartments of the cell, with no obvious nuclear or cytoplasmic exclusion (Fig. 4A). Furthermore, treatment of cells with hydrogen peroxide did not alter this diffuse cellular localisation pattern (Fig. 4A). Thus, Crr1 has a distinct cellular localisation pattern to that previously documented for the Ssk1/Mcs4 and Skn7/Prr1 response regulators in *S. cerevisiae* and *S. pombe* and, moreover, this pattern is not affected by oxidative stress or mutation of the predicted phospho-aspartate residue.

To further investigate whether two component-mediated phosphorylation of Crr1 is important for the function of the protein, the hydrogen peroxide sensitivities of *crr1Δ* cells expressing either Crr1-GFP or Crr1^D/N^-GFP were compared. Strikingly, cells expressing Crr1^D/N^-GFP were found to be reproducibly more sensitive to hydrogen peroxide than the isogenic ‘wild-type’ cells expressing Crr1-GFP and, moreover, displayed similar sensitivity to *crr1Δ* cells (compare Figs. 2 and 4B). This phenotype associated with Crr1^D/N^-GFP was replicated in SN148 cells (Fig. S2C). Based on the mutational analyses of other two component signal transduction proteins in bacteria and fungi these results strongly suggest that two component-mediated phosphorylation of Crr1 is important for the function of the protein in contributing to hydrogen peroxide resistance in *C. albicans*.

### Crr1 is not required for *C. albicans* virulence

**Figure 5:**
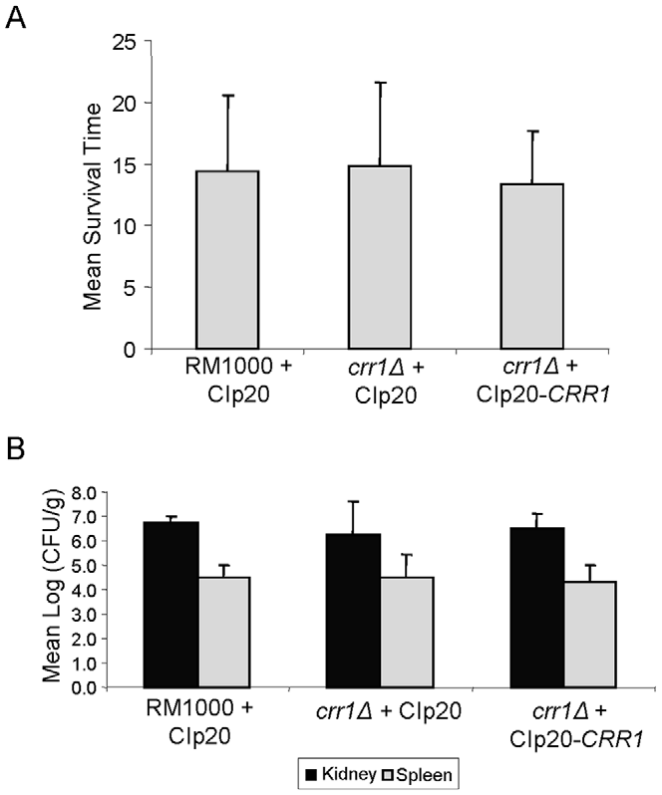
Crr1 is dispensable for the virulence of *C. albicans*. (A) Mean survival times, and (B) organ fungal burdens, for BALB/c mice infected with either WT (RM1000+Clp20, JC806), *crr1Δ* (*crr1Δ*+Clp20, JC566) or *crr1Δ* reintegrant (*crr1Δ*+Clp20-*CRR1*, JC803) cells following the standard 28-day survival murine model of systemic candidiasis.

As shown above, Crr1 is required for wild-type levels of oxidative stress resistance in *C. albicans*. Interestingly, loss of Ssk1, but not Skn7, influences the virulence of *C. albicans* despite the observations that both are implicated in the oxidative stress response [24], [42]. Hence, we examined the potential role of Crr1 in virulence using the standard 28 day murine model of systemic candidiasis. Isogenic wild-type (JC806), *crr1Δ* (JC566), and reintegrant *crr1Δ*+*CRR1* (JC803) strains, which all express *URA3* from the *RPS10* locus, were tested in the murine model of systemic candidiasis. Such controls are necessary as it is well-established that the genomic location of the *URA3* disruption marker can influence expression levels which significantly impacts on the virulence of *C. albicans* [43]. However, deletion of *CRR1* was found to have no detectable impact on the virulence of *C. albicans* (Fig. 5A). Mice infected with the *crr1Δ* mutant had a mean survival time of 14.8±6.7 days compared with 14.3±6.2 and 13.3±4.3 days for mice infected with wild-type or reintegrant *crr1Δ*+*CRR1* cells, respectively. Kaplan-Meier and log rank tests showed no difference in virulence between the strains (*P* = 0.877). Consistent with these conclusions, no statistically significant difference in either kidney (*P* = 0.314) or spleen (*P* = 0.782) fungal burdens from mice infected with wild-type, *crr1Δ* or *crr1Δ*+*CRR1* reintegrant cells was detected (Fig. 5B). Hence, in contrast to the *C. albicans* Ssk1 response regulator [42], we find no evidence that Crr1 is involved in the virulence of this fungal pathogen using the mouse model of systemic candidiasis.

## Discussion

Here we have identified and characterised a novel response regulator in *C. albicans* which we have named Crr1. Two component signal transduction pathways are utilised to respond to environmental conditions in fungi and Crr1 was found to be important for the response of *C. albicans* to hydrogen peroxide. Furthermore, mutant cells expressing Crr1 in which the predicted phospho-aspartate was mutated to asparagine also displayed increased sensitivity to hydrogen peroxide, indicating two component-mediated phosphorelay is important for Crr1 function. Notably, extensive phylogenetic analyses revealed that this previously uncharacterised response regulator is solely found in fungi belonging to the *Candida* CTG clade [38]. Thus, these data suggest that the Crr1 family is involved in the response of cells to oxidative stress within a specific subgroup of fungal species.

*C. albicans* also contains members of the ubiquitous Ssk1 and Skn7 families of response regulator proteins which are present in diverse fungal species in addition to the *Candida* CTG clade [44]. It is intriguing that all three response regulators, Crr1, Skn7 and Ssk1, are required for oxidative stress resistance in *C. albicans* [24], [25]. Whilst Skn7 likely directly mediates the expression of antioxidant encoding genes, Ssk1 has been shown to be required for the hydrogen peroxide-induced activation of the Hog1 SAPK [25]. Here, our analysis of cells lacking *CRR1* revealed that Hog1 activation is not impaired in *crr1Δ* cells. However, as *ssk1Δcrr1Δ* cells were no more sensitive to hydrogen peroxide than either single mutant, this suggests that Crr1 and Ssk1 may act in the same pathway. Thus, whilst Ssk1 functions upstream of Hog1, Crr1 may function downstream of this SAPK. Clearly, the nature of the relationship between Ssk1 and Crr1 function in the response of cells to hydrogen peroxide requires further investigation.

Although the receiver domain located in the C-terminal region of Crr1 contains all of the key residues required for the function of this domain, sequence analysis of the N-terminal region of Crr1 provided no insight into the potential function of this response regulator protein. Furthermore, this analysis was not facilitated by defining the localisation of Crr1, which is found throughout the cell. However, the open reading frame encoding Crr1 (*orf19.5843*) was previously identified in transcript profiling studies as a gene whose expression was up-regulated in the absence of the adenylyl cyclase Cdc35 [45], or in a conditional phospholipase C mutant at elevated temperatures [46]. Nonetheless, an extensive analysis of *crr1Δ* cells failed to establish a link between Crr1 and any cAMP- [45], [47] or phospholipase C- [46] dependent processes in *C. albicans* (Fig. 2). Interestingly, a recent report linked cAMP-mediated signalling to oxidative stress resistance in *C. albicans*, as the quorum sensing molecule farnesol stimulates resistance to hydrogen peroxide by inhibiting the Ras-cAMP pathway [48]. However, similar increases in farnesol-induced hydrogen peroxide resistance were observed in both *crr1Δ* (6.9%) and *crr1Δ*+*CRR1* (8.9%) reconstituted cells to those reported previously [48]. Thus, these data indicate that Crr1 mediates the resistance of *C. albicans* to hydrogen peroxide independently of both Hog1 activation and farnesol-mediated inhibition of cAMP signalling.

In this paper we describe the identification of a response regulator protein that appears to be confined to the *Candida* CTG clade [38]. All of the Crr1-related proteins in the haploid and diploid members of the clade share extensive homology between their receiver domains suggesting that they had a common ancestor (Figs. 1B; S1). In addition, we have identified at least two conserved regions within the N-terminal regions of the diploid members of the Candida clade (Fig. 1B) although the potential function(s) of these regions awaits further investigation. Given that two component signal transduction pathways are utilised to respond to the environment by regulating appropriate cellular responses it is tempting to speculate that the diploid members of the *Candida* CTG clade respond to the oxidising agent hydrogen peroxide in a similar manner through the function of these Crr1 homologues. It is important to note, however, that whilst all members of the *Candida* CTG clade can cause disease in humans [49], we can find no evidence that Crr1 affects the virulence of *C. albicans* in a standard mouse model of systemic candidiasis. Thus, it is possible that Crr1 functions to allow adaptation to an environmental niche outside the human host. Alternatively, Crr1 function may be required for *C. albicans* to exist as a commensal organism within specific host niches that are not replicated in a systemic model of disease. In this regard it is noteworthy that a recent study revealed that the expression of *CRR1* in *C. albicans* is induced during the late stages of biofilm formation [50]. Clearly, much is still to be learnt about the biological roles of the novel Crr1 response regulator, which is only present in the *Candida* CTG clade of fungal species.

## Supporting Information

Figure S1 **Sequence analysis of the closest homologues of CaCrr1 in the haploid members of the** ***Candida*** **CTG clade.** (A) Clustal alignment of CaCrr1 with the closest homologues of CaCrr1 present in *Debaromyces hansenii* (DEHA2G23386g), *Candida guilliermondii* (PGUG_04093) and *Candida lusitaniae* (CLUG_02461). The main shared region of homology is limited to the potential receiver domain located in all of these proteins. Residues that are identical between all four proteins are indicated by bold, the aspartate and lysine residues conserved in all receiver domains are shown in red bold, and the aspartate residue which is predicted to be phosphorylated by two component signal transduction by a bold red “P”. Note that the homology between the receiver domains extends to the replacement of amino acids with others with similar chemical properties. A colon indicates a highly similar substitution and a full stop a similar substitution. (B) Clustal alignment of the closest homologues of CaCrr1 present in *D. hansenii* (DEHA2G23386g), *C. guilliermondii* (PGUG_04093) and *C. lusitaniae* (CLUG_02461) revealed that the main region of homology shared between proteins in the haploid group in the *Candida* clade is limited to the potential receiver domain located in all three proteins. Residues shared by all three proteins are highlighted as described in (A) above. The predicted protein sequences of the Crr1 homologues in the haploid members of the *Candida* clade were obtained by BLAST analyses at the *C. albicans* genome web site (http://candidagenome.org/).
(TIFF)

Figure S2 **Phenotypic analysis of Crr1 function in the SN148** ***C. albicans*** **background, replicates that in RM1000 cells.** (A) SN148 cells lacking *CRR1* are sensitive to hydrogen peroxide but not other compounds. Approximately 10^3^ cells, and 10-fold dilutions thereof, from exponentially-growing WT (SN148+CIp30; JC747), *crr1Δ* (JC1572) and *crr1Δ*+*CRR1* (JC1574) strains were spotted onto YPD plates containing the indicated agents. Plates were incubated at 30°C for 24 h. (B) Ssk1 but not Crr1 is required for Hog1 activation in response to hydrogen peroxide in SN148 cells. Western blot analysis of whole cell extracts isolated from wild-type (WT, JC747), *ssk1Δ* (JC1552), *crr1Δ* (JC1572), and *crr1Δ*+*CRR1* (JC1574) cells after treatment with 5 mM hydrogen peroxide or 1 M NaCl for the specified times. Western blots were probed with an anti-phospho-p38 antibody, which specifically recognises the phosphorylated, active form of *C. albicans* Hog1 (Hog1-P). Total levels of Hog1 protein were determined by stripping the blot and reprobing with an anti-Hog1 antibody which recognises both phosphorylated and unphosphorylated forms of Hog1 (Hog1). (C) Mutation of the putative phospho-aspartate of Crr1 impairs hydrogen peroxide resistance in SN148 cells. 10^3^ cells, and 10-fold dilutions thereof, of exponentially-growing *crr1Δ* cells expressing either *CRR1-GFP* (JC1576) or *CRR1^D/N^-GFP* (JC1578) were spotted onto YPD plates containing the indicated concentrations of hydrogen peroxide and incubated at 30°C for 24 h.
(TIFF)
