# Supplementary figures and images for: Identification of a Novel Response Regulator, Crr1, That Is Required for Hydrogen Peroxide Resistance in Candida albicans
Source: PLoS One. 2011 Dec 2;6(12):e27979. doi: 10.1371/journal.pone.0027979 (PMC3229506; doi:10.1371/journal.pone.0027979)

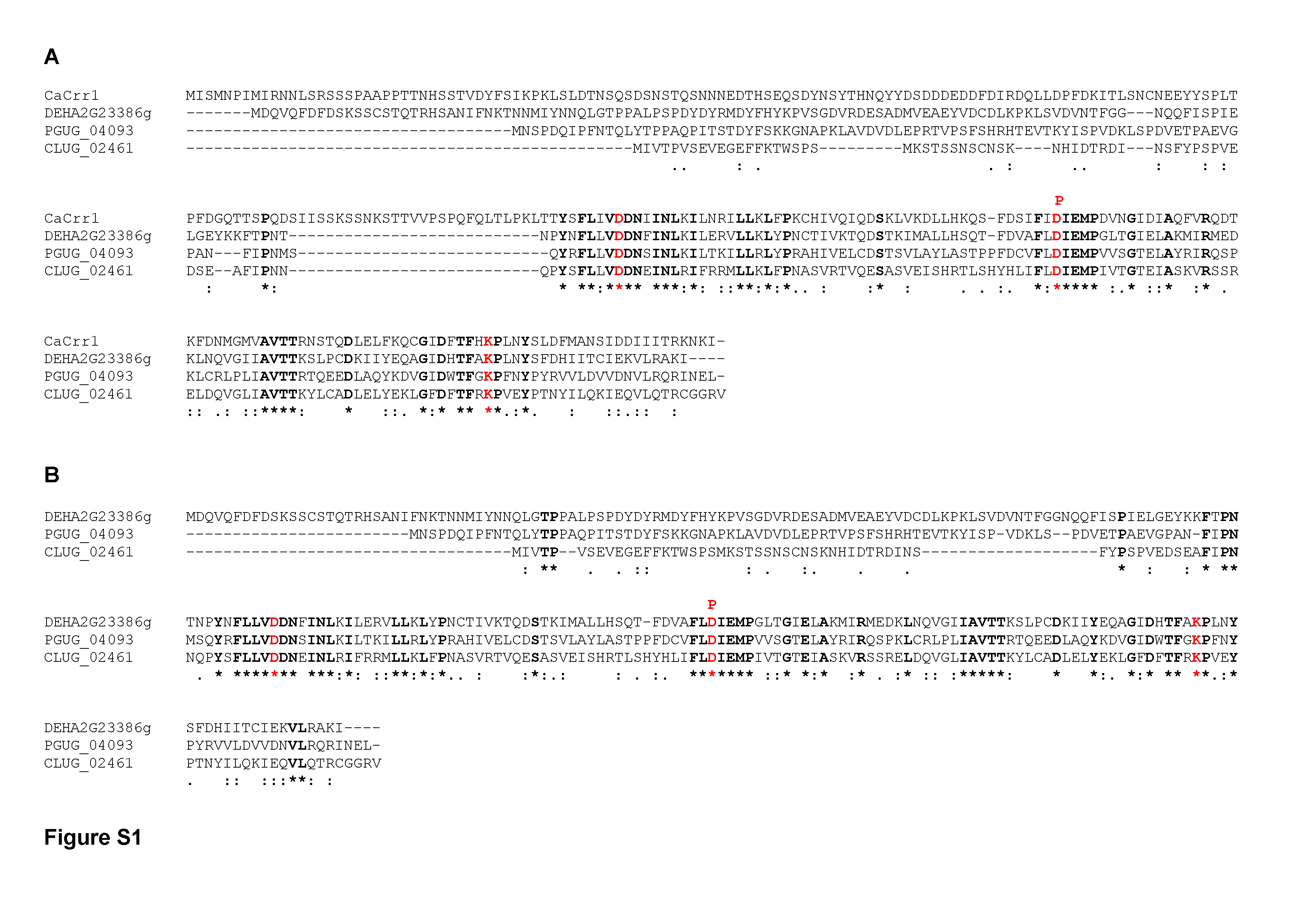

Supplement: Figure S1 — Sequence analysis of the closest homologues of CaCrr1 in the haploid members of the Candida CTG clade. (A) Clustal alignment of CaCrr1 with the closest homologues of CaCrr1 present in Debaromyces hansenii (DEHA2G23386g), Candida guilliermondii (PGUG_04093) and Candida lusitaniae (CLUG_02461). The main shared region of homology is limited to the potential receiver domain located in all of these proteins. Residues that are identical between all four proteins are indicated by bold, the aspartate and lysine residues conserved in all receiver domains are shown in red bold, and the aspartate residue which is predicted to be phosphorylated by two component signal transduction by a bold red “P”. Note that the homology between the receiver domains extends to the replacement of amino acids with others with similar chemical properties. A colon indicates a highly similar substitution and a full stop a similar substitution. (B) Clustal alignment of the closest homologues of CaCrr1 present in D. hansenii (DEHA2G23386g), C. guilliermondii (PGUG_04093) and C. lusitaniae (CLUG_02461) revealed that the main region of homology shared between proteins in the haploid group in the Candida clade is limited to the potential receiver domain located in all three proteins. Residues shared by all three proteins are highlighted as described in (A) above. The predicted protein sequences of the Crr1 homologues in the haploid members of the Candida clade were obtained by BLAST analyses at the C. albicans genome web site (http://candidagenome.org/). (TIFF) [file pone.0027979.s001.tiff]

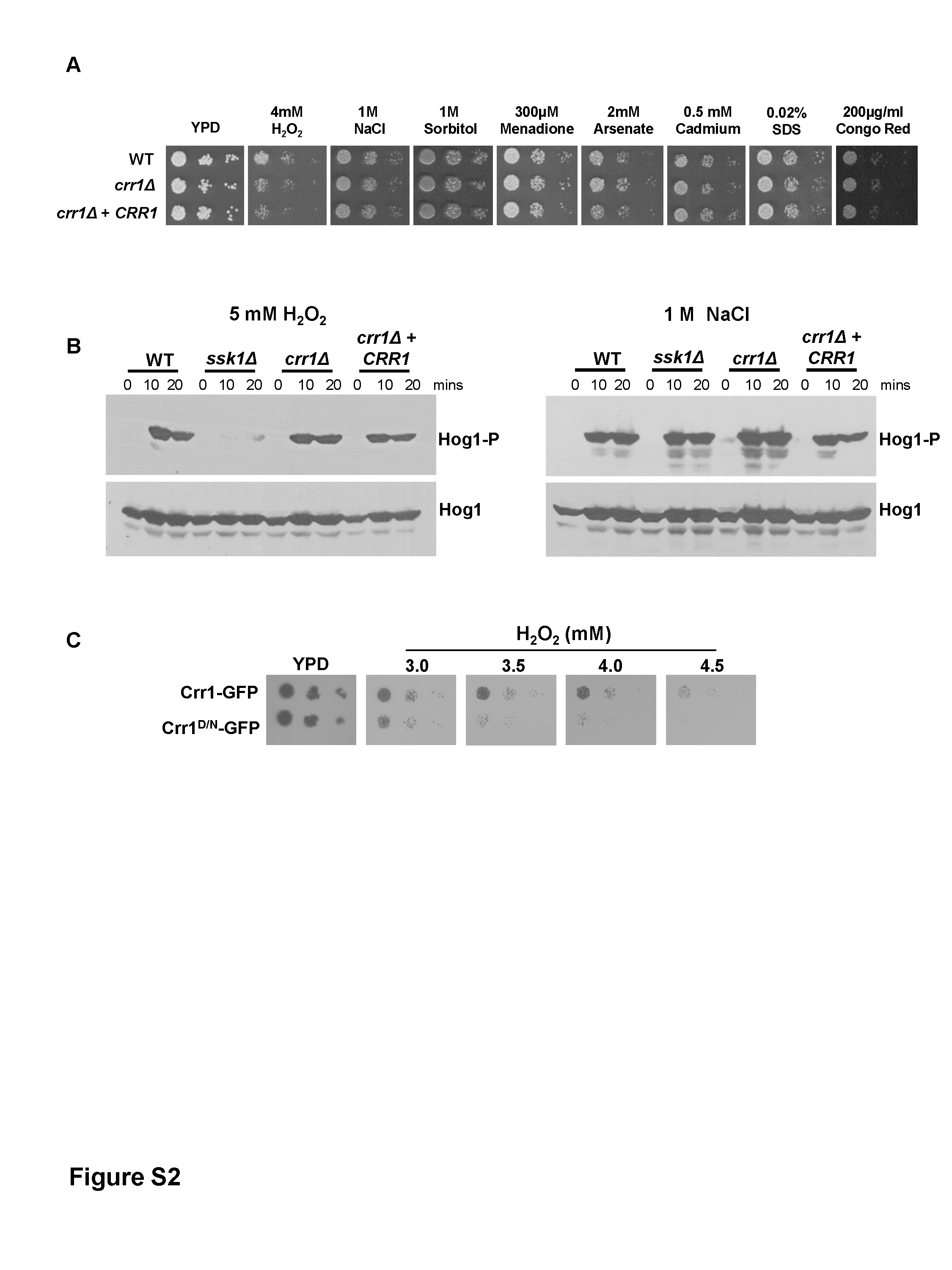

Supplement: Figure S2 — Phenotypic analysis of Crr1 function in the SN148 C. albicans background, replicates that in RM1000 cells. (A) SN148 cells lacking CRR1 are sensitive to hydrogen peroxide but not other compounds. Approximately 103 cells, and 10-fold dilutions thereof, from exponentially-growing WT (SN148+CIp30; JC747), crr1Δ (JC1572) and crr1Δ+CRR1 (JC1574) strains were spotted onto YPD plates containing the indicated agents. Plates were incubated at 30°C for 24 h. (B) Ssk1 but not Crr1 is required for Hog1 activation in response to hydrogen peroxide in SN148 cells. Western blot analysis of whole cell extracts isolated from wild-type (WT, JC747), ssk1Δ (JC1552), crr1Δ (JC1572), and crr1Δ+CRR1 (JC1574) cells after treatment with 5 mM hydrogen peroxide or 1 M NaCl for the specified times. Western blots were probed with an anti-phospho-p38 antibody, which specifically recognises the phosphorylated, active form of C. albicans Hog1 (Hog1-P). Total levels of Hog1 protein were determined by stripping the blot and reprobing with an anti-Hog1 antibody which recognises both phosphorylated and unphosphorylated forms of Hog1 (Hog1). (C) Mutation of the putative phospho-aspartate of Crr1 impairs hydrogen peroxide resistance in SN148 cells. 103 cells, and 10-fold dilutions thereof, of exponentially-growing crr1Δ cells expressing either CRR1-GFP (JC1576) or CRR1D/N-GFP (JC1578) were spotted onto YPD plates containing the indicated concentrations of hydrogen peroxide and incubated at 30°C for 24 h. (TIFF) [file pone.0027979.s002.tiff]
